# Vomiting: The Action and Use of Emetics

**Published:** 1843-01

**Authors:** 


### ART. V.

1. *Das Erbrechen; die Wirkung und Anwendung der Brechmittel.* Von J. W. ARNOLD, Prof. in Zurich, &c.—*Stuttgart,* 1840. 8vo, ss. 403.
*Vomiting: the Action and Use of Emetics.* By Dr. ARNOLD.—*Stuttgart,* 1840.

2. *Die Lehre vom Erbrechen; Nach Erfahrungen und Versuchen.* Von Dr. JULIUS BUDGE, &c. *Mit einer Vorrede von* Dr. FRIEDRICH NASSE.—*Bonn,* 1840. 8vo, ss. 243.
*The Doctrine of Vomiting, from Observations and Experiments by* Dr. BUDGE. *With a Preface, by* Dr. NASSE.—*Bonn,* 1840.

IN the works whose titles head this article the physiology, pathology, therapeutical indications and uses of vomiting are amply and minutely discussed. Of necessity there is much matter introduced and many facts and experiments recapitulated which are familiar to our readers. These we shall omit; and shall confine our notices to points recommended by novelty or practical importance. The former treatise being the more complete, we shall direct our attention in the first place and principally to it, making such occasional references to that of Dr. Budge as we may find necessary.

Dr. Arnold, after a description of the phenomena of vomiting, proceeds to lay before his reader an historical view of the opinions entertained on the subject, from the earliest periods to the present. We do not find that it would be peculiarly entertaining or useful to introduce here a series of doctrines and reputed facts which, besides being commonly known, have been either exploded by more recent and accurate researches, or else explained in a manner different from that of the original observers.

*The mechanism of vomiting.* As Dr. Budge justly remarks, (p. 132,) to treat of this subject in all its relations, would imply a "perambulation" through the whole domain of pathology; and he might have added, of physiology also. In the case of certain animals, vomiting is a strictly natural or normal process. Several species of polypi get rid of their excrements by vomiting. Bees in this manner excrete their honey. Many fishes and birds—as carps, barbels, pikes, herons, eagles, falcons, crows —vomit the feathers and hair of the animals they prey on. Some men ruminate, which is a kind of vomiting; others vomit with great facility. Dr. Budge is of opinion that the frequent rejection, by children, of the mother's milk, when infants have oversuckled themselves, is not to be regarded as a morbid process, since it is performed without the slightest effort or disgust; the little creatures, as the author observes, oftentimes laughing in the very act. (Budge, p. 1.)

Three principal views have been entertained as to the mechanism by which the expulsion of the contents of the stomach upward is effected. Magendie is or was of opinion that the stomach is passive in the act; and that the contraction of the diaphragm and abdominal muscles on that organ is the sole cause of the phenomenon. This view was adopted by Richerand, Rostan, Piedagnel; while Marquais, Maingault, Portal, Tantini, Drs. Graves, Stokes, and Hall have embraced, more or less, contrary opinions. Maingault reports experiments in some of which the abdominal muscles were cut away; in others the nerves of the diaphragm were bisected and the muscle itself removed, and still vomiting took place. He attributes vomiting exclusively to a gradual anti-peristaltic movement and contraction of the stomach. Portal's observations led him to conclude that vomiting takes place during expiration; consequently, during the relaxation of the diaphragm. The non-essentiality of the action of the diaphragm to the production of vomiting seems to be decisively proved by the case of Drs. Graves and Stokes, namely, that of a man in whose disease vomiting was a principal symptom, but in whom the stomach was found situated superiorly to the diaphragm, its vomitive efforts, consequently, could not have in any degree depended on the contraction of that muscle. It is, moreover, to be observed that certain birds which have no diaphragm are yet subject to vomiting, or rather, perform it as a natural process.

Dr. Hall's rationale of vomition is as follows: 1, During the act the larynx is closed; in consequence of which, 2, The diaphragm and its apertures are relaxed; 3, All the muscles of respiration are called into action in the production of vomiting; 4, But active expiration being impracticable, owing to the closure of the larynx, and therefore all the action of the expiratory muscles being converged on the stomach, vomiting necessarily ensues. As from the relaxation of the diaphragm the thoracic and abdominal cavities are converted into one, it matters not on which side of the diaphragm the stomach is situated. This explanation of Dr. Hall partially reconciles, in Arnold's opinion, the case above quoted of Drs. Graves and Stokes with the views of Magendie.

The experiments of Béclard and Legallois, undertaken at the request of the Society of the School of Medicine of Paris, lead to the following medium views. The stomach, under emetic stimulation, exhibits a gradual and alternate relaxation and contraction near the pylorus and about two inches from it, and even at the organ's middle part, but without vomiting ensuing, provided the emetic excitement produces no further effects than those detailed. If, however, the excitement referred to proceeds further, the œsophagus begins to play an important part: grasping and convulsively carrying upwards the stomachic contents within its reach. But this action of the œsophagus produced vomiting only when the stomach was full of fluid. On cutting the diaphragm also, vomiting could be excited only when the stomach was full. Moreover, on making a section of the abdominal muscles vomiting could be made to take place only when the stomach was kept under the ribs and was full of fluid, otherwise the efforts of the diaphragm and œsophagus were inadequate to the effect. Béclard's general conclusions are, that the stomach is not so influential in the production of vomition as was formerly supposed, but that is not entirely inefficient; that from it goes forth the "sympathetic excitement" which gives rise to the peculiar contractions of the abdominal muscles and of the diaphragm which accompany vomiting and in part cause it; that the œsophagus plays a principal part in the process. Finally, he divides the act of vomition into two stages or periods, in the former of which the stomach, though in movement cannot throw off its contents without external support, as of the abdominal muscles, hand, &c., but during which interval the œsophagus is convulsively acting on the stomach as if to draw within its grasp the contents of that organ; in the second period these contents, brought within the reach of that tube, are expelled by its independent efforts: efforts which the diaphragm and abdominal muscles do nothing to aid or to maintain.

Dr. Arnold seems to hold very nearly the same views as those now detailed. He does not appear, however, to attach quite so much importance to the œsophagus as an agent in vomition as Béclard appears to do, since he remarks, (p. 90,) that "a very free and rapid vomiting is possible only when the œsophagus is *passive* and opposes no resistance to the impelling cause which brings about the vomitive crisis." Hence it appears that he infers the possibility of that tube being entirely inert during the act.

Dr. Arnold holds (p. 91) that it is only at the beginning of vomition, only during the inspiration which marks the commencement of that act, and while as yet no ejection has taken place, that the diaphragm is in a state of contraction; but that the actual expulsion of the stomachic contents happens only during relaxation of that muscle; *the abdominal muscles* meantime *remaining contracted.*

Dr. Budge informs us that his conclusions were founded on numerous experiments made on himself and others, and also on persons under disease. (Budge, p. 3.) He observes that children whose stomachs are more muscularly energetic than those of adults vomit more readily than these; and that while diseases of the stomach are very frequently accompanied by vomiting, those of the diaphragm and abdominal muscles are seldom so. But in regard to the value of the fact, (as brought to bear against Magendie's views,) that birds which want the diaphragm, yet possess the power of vomiting, he observes that it must hence be not inferred that in them the stomach alone effects vomiting. The muscles which conduce to the respiratory act must, he alleges, cooperate. This, he asserts, is not the case with quadrupeds; in which, by stimulating the pylorus, he has been able to produce vomiting, even when the stomach was withdrawn from the abdominal cavity; although never, in similar circumstances, could he excite it in birds. (Budge, p. 24.)

Dr. Budge's opinion of the power exerted by the stomach in vomiting seems to be somewhat different from that of Arnold. We shall not detail, in all their variety and minuteness, the experiments instituted by the author, but shall relate the chief circumstances of one experiment, by which the power of the stomach to rid itself of its contents, in the way of vomiting, without the aid of the diaphragm and abdominal muscles, seems to be proved. The belly of a dog was incised, and the stomach withdrawn from the abdominal cavity to such an extent as to allow of its pyloric and cardiac ends being visible. The pylorus was then tied. Some soup—but whether emeticized is not stated—was then poured down the animal's gullet. The phenomena which principally relate to the subject of this article were are as follows: Soon after ingestion of the soup the stomach swelled to three times its usual size, and appeared like a greatly distended bladder. Eight minutes after the animal became very restless, and though tied made violent efforts to press its stomach against the table on which it lay. These efforts Dr. Budge thinks were due to the same instinctive impulse which leads us, when under the action of an emetic, to press the hands upon the abdomen. The pyloric end of the stomach now contracted itself and became hard: all the rest of the stomach appeared distended. The pyloric portion made efforts to eject or toss its contents to the left extremity of the organ, and downwards to the great curvature. The contraction of the pylorus and the expansion of the stomachic cavity were simultaneous, and so rapid as to require close attention on the part of the observer, in order to note accurately the precise nature of the movements. With every such convulsion of the stomach there was usually complicated borborygmus, from the air being driven from the right to the left of the organ. As soon as the spasmodic effort of the pylorus terminated, the other part of the stomach lost its distension, and at the same moment eructation or actual vomiting ensued: eructation, if the convulsive act of the pylorus and and the contraction of the stomach had been moderate and gradual—vomiting if they had been energetic and abrupt. On the stomach being replaced in the abdominal cavity, vomiting took place much more readily and easily. Dr. Budge withdrew it again, and placed his outspread hand upon it, but without exercising any pressure; and on fluid being anew introduced into the organ he felt the latter bearing against his hand. Exerting now considerable pressure on the stomach, vomiting took place easily; and the following fact is worthy of attention, as illustrative of the sympathetic excitement which, emanating from the stomach as a centre, extends to all the respiratory apparatus when nausea is felt. Even when the stomach was lying external to the abdominal cavity, and could consequently no longer operate, from its bulk, as a stimulus to the abdominal muscles; these muscles yet contracted simultaneously with every vomitive effort, and relaxed themselves immediately on that effort terminating.

Dr. Budge infers that the stomach may, under certain circumstances, effect vomiting independently of the diaphragm and abdominal muscles; but that in natural and ordinary circumstances these muscles always cooperate with that organ (p. 34); and at p. 24, after referring to the experiments of Maingault and Béclard, he further concludes that when the stomach is in its natural situation there is no act of vomiting to which the diaphragm and abdominal muscles do not contribute: that, on the other hand, the stomach itself is never inert, but that the stomach and the muscles always mutually cooperate; that not only the stomach but even the whole tract of the intestinal canal sympathises with the peculiar affection of the nervous centres from which vomiting arises, is proved by the appearance, in cases of long and obstinate vomition, of fæcal matters in the egesta.

It may here be noticed, that the peculiar action of the stomach under emetic excitement consists of a general and gradual diminution of its volume, having something, so far as we have observed, of a *rhythmic* character.

Dr. Budge informs us (p. 25,) that dogs and cats are the most favorable subjects for experiment: that rabbits can be made to vomit only with the greatest difficulty. Dogs vomit more easily than cats. Indeed, from the experiment related at p. 38, and the remark which closes it, (p. 40,) we are led to infer that Dr. Budge generally failed in producing vomiting in the cat after the stomach was withdrawn from the abdominal cavity.

The singular distension of the stomach which characterizes a state of nausea and seems invariably to accompany vomition, is noticed by

Magendie. "I was not," observes this physiologist, "a little surprised to see the stomach become filled with air, in proportion as the nausea approached. One could scarcely be deceived in this matter, since the organ at least tripled its volume. . . . . . . . . The organ disembarrassed itself of air and of a portion of aliment: but immediately after the exit of these matters it was loose, and it was not till after some seconds that, contracting itself by little and little on itself, it reassumed the same dimensions which it had before the vomiting."

Magendie seems wholly to attribute this singular phenomenon to the entrance and presence of air. But Dr. Budge, with more accuracy, remarks, that the entrance of air is a consequence of the distension, not the cause of it. He designates the distension "an active movement or exertion" (bewegung) of the organ, and thinks it in a great measure due to the contraction of the pylorus, on the one hand, and of the œsophagus, on the other. (p. 41.)

*Causes of vomiting in man.* The states of the body which favour or may occasion vomiting are treated of by Dr. Arnold at p. 95 et seq. The first of these is the figure of the stomach itself. Schultz would have it that the reason children vomit so much more readily than adults is, that their stomachs resemble more in form those of carnivorous brutes; while the stomachs of adults approach more to those of rabbits, hares, &c. A better explanation is found in the fact that the stomachs of children are more conical towards each extremity: the œsophagus is inserted into the fundus itself; the great curvature has, at this early period of life, a very obtuse curve, and runs parallel to the small one. The stomachs of adults are, on the other hand, more rounded; the œsophagus no longer enters, as formerly, in the left end or the fundus, but in the space between the left end and the pylorus these two outlets approach greatly: the small curvature is consequently rendered very acute and short; the great curvature has undergone proportional extension and increased obtuseness of its curve. In children, consequently, the peristaltic and antiperistaltic movements, and the transmission of the stomachic contents in either direction are more easy than with adults—the stomach having only one axis, as it were, in infants, but two in adult life. (Arnold, p. 100.) The *nervous* mechanism of vomiting we shall come to presently.

Among the states of the body which may give rise to vomiting are hypertrophy of the mucous or sub-mucous membrane, or of the muscular coat of the stomach; carcinomatous disease of that viscus; inflammation of the mucous membrane, as pointed out by Andral. In exanthematous diseases, vomiting is a common symptom. In connexion with pemphigus, Gilibert has noticed vesicles in the stomach; and in fatal smallpox, pustules have been observed there: aphthæ are also frequently developed in it; yet in none of the above cases is the occurrence of vomiting to be regarded as necessarily indicative of the existence of the appearances referred to in the stomach, since often in the above-named diseases energetic vomiting is present without the lesions alluded to.

A high degree of excitability of the stomach, a nervous gastralgia, a lesion peculiarly dynamical and neither depending on or leading to any structural change, is a frequent cause of vomiting, although there are forms of dynamic gastralgia in which there is not only no vomiting, but on the contrary, a great increase of digestive vigour. The sensibility of the stomach and its motor power depending on different nerves, there may be pain without vomiting, and vomiting without pain or even nausea. The organ owes its sensibility to the pneumogastric, its motor power to the spinal and accessory and in part to the ganglionic nerves; and hence separate affections of these nerves may produce separate and distinct phenomena; though frequently lesions of the one class of nerves sooner or later involve like consequences in the other.

Alteration of the stomach's secretions, or the presence of abnormal fluids there, as of bile, blood, or metastatic excretions, urinary or uterine; obstruction of the intestinal tube by polypous growths, intus-susception, or hernia; disease of contiguous viscera, as pressure of an enlarged liver, spleen, or loaded colon; or inflammation or calculous irritation of the kidney—may severally occasion vomiting. In the last case, the stomachic excitement may be due to the irritation of the renal plexus extending to the solar or to the vagus, which contributes to the formation of the latter plexus; that irritation being transmitted by the vagus to the medulla oblongata; from which convulsive motory phenomena may be set up in the diaphragm and abdominal muscles. The irritation may moreover be propagated from the renal plexus by the medium of the lowest thoracic nerve to the spinal cord, inasmuch as that plexus is reinforced from the nerve just named.

All violent respiratory efforts may induce vomiting, as excessive hiccuping, coughing, profound sobbing, shrieking, stentorian singing or shouting. This effect is partly due to the circumstance of the respiratory and digestive organs being acted on by the same muscles, partly to their sensibility being derived from the same nerve: and as irritation in the stomach often produces cough by the reflex circuit of the medulla oblongata, so, by the same channel, may cough or any other pulmonary convulsion produce vomiting.

Diseases of the nervous centres often set in with vomiting. This is more especially the case when the medulla oblongata is the site of the morbid affection; and when this happens there is usually contemporaneous respiratory symptoms, consisting of cough, hiccup, &c. Morbid conditions of various parts of the nervous centres, besides the medulla oblongata—as the spinal cord, cerebrum and cerebellum, pons, fornix, corpora quadrigemina, corpora striata—excite vomitive efforts, but not in equal degrees. Abnormal states of the cerebellum produce neither frequent nor violent phenomena of this sort. According to Schwartz, an anti-peristaltic action of the stomach ensues from pressure on this portion of the brain. Burdach affirms that constipation, involuntary evacuations, and vomiting are more frequently caused by disease of the cerebellum; but pure gastritic symptoms and diarrhœa more commonly by affections of the cerebrum. According to the same physiologist, vomiting takes place more remarkably from abnormal states of the pons, than from those of the medulla oblongata, cerebrum, or cerebellum. In disease of the fornix vomiting is not a frequent or remarkable symptom. On the other hand, it ensues more commonly from lesions, first, of the corpora quadrigemina, and next of the ventricles, than from those of the cerebrum; from which facts Burdach infers that the corpora quadrigemina stand in the nearest relation to the digestive organs and functions, and that from them the common sensibility and the assimilative power of the stomach are derived. Dr. Arnold is of opinion that while Burdach somewhat overrates the degree of connexion, it is not to be doubted that there is a certain relation between the cerebral parts above named and the stomach, and that it is in the corpora quadrigemina that sensations in the latter organ become subjects of *perception* or consciousness. Next to the fornix these *corpora* are the parts of the brain the abnormal states of which most remarkably produce vertigo, terminating in vomiting. (Arnold, pp. 155-6.)

Dr. Arnold's account of the manner in which lesions of the nervous centres produce vomiting is as follows. (p.157.) As it is characteristic of these centres to act eccentrically or peripherally when any excitement is applied to an external part or organ, so when the stomach, which, in relation to these centres, is to be viewed as an external or peripheral organ, is subjected to any irritation, a reactive influence goes forth from the nervous centres, the vital object of which is to oppose or subdue that irritation. "Now," proceeds the author, "as the medulla oblongata, which commands (beherrscht) the spinal cord and its nerves and also those nerves distributed to the abdominal and thoracic muscles, which, further, receives through the vagus the sensations from the organs of the breast and belly, and which gives rise to the involuntary movements in these organs, through the phrenic and accessory as well as through the spinal nerves, so may excitable states of the medulla oblongata occasion such movements, in like manner as if they were occasioned by a stimulus acting on the digestive or respiratory organs." We presume that the author means to teach that while direct irritation of the stomach may so affect the medulla oblongata as to cause it to send forth reflex influences leading to vomition, so, on the other hand, original disease of the medulla oblongata may cause vomiting without any direct irritation of the digestive organs. This is a universally admitted fact, of which our readers would deem it supererogatory in us to adduce lengthened proof.

Irritable or excited states of the organs of special sense may occasion vomiting, as disagreeable odours or tastes, &c. This effect is not explicable on the mere ground of nervous connexion between the organs of sense, now named, and the stomach, but is due to the peculiar relation in which the former stand to the latter, destined as they are to take cognizance of the saporous and odoriferous qualities of substances previous to their being permitted to pass into the stomach, and therefore possessing similar sensibilities, with the digestive organs, and sharing its likings and dislikings. The vomiting which follows, occasionally, from acute or unpleasant sounds, is a neuro-sympathetic affection. It occurs in consequence of the auricular branch of the vagus, conveying excitement to the medulla oblongata, from which a "reaction" goes forth to the various organs supplied by the vagus. Hence follows cough, in the event of the lungs being more affected; vomiting, in the event of the stomach being so.

Affections of the eyes, more particularly irritation of the retina, sudden light, or rapid interchanges of light and darkness, and quick motions in objects looked at, cause vomiting, from the circumstance that such impressions of the visual nerves affect the same parts of the brain as those which produce vomiting, namely, the quadrigemina.

At page 160, Dr. Arnold explains the share which the vagus, accessory, and phrenic nerves have in vomiting. As it is through the vagus that abnormal states of the stomach which lead to vomiting are, when sensible, perceived by us; and by the same channel, that irritating causes in that organ operate on the medulla oblongata, it is obvious that this nerve must have an important influence in vomition. Yet animals vomit readily after section of the pneumo-gastric nerve. Thus, in dogs, this nerve being divided, mere barking, running, or twisting of the body is sufficient to cause vomiting. But the apparent anomaly of this case is owing simply to the action of the diaphragm and abdominal muscles; the stomach being passive. The vomiting takes place still more readily, if the accessory nerve is divided at the same time with the vagus; since, thereby, the œsophagus is paralysed, and consequently opposes no resistance to the escape of the stomachic contents. On the other hand, irritation of the accessory nerve will cause vomiting by producing anti-peristaltic movements in the stomach and œsophagus.

Irritation of the phrenic nerve may cause vomiting, by producing violent contractions of the diaphragm; and when hiccup accompanies any gastric or thoracic affection, we may infer that the diaphragm is implicated. And, on the other hand, we may conclude that any affections of the nervous centres which give rise to vomiting and hiccup, have most probably their seat in the medulla oblongata, cerebellum, or corpora quadrigemina. (Arnold, p. 162.)

As to the part, if any, which the ganglionic system plays in vomiting, physiologists are not agreed. Valentine supposes that irritation of the ganglia corresponding to the œsophagus, stomach, and intestines causes contractions of these parts. Budge holds that neither the sympathetic or the cæliac ganglion is the ultimate source of the intestinal movements, but that these nervous parts derive their power from the spinal cord. The cæliac ganglion he regards as having no other office than that of a non-conductor, or "mechanical obstruction" placed on the nerve, to prevent too great sensibility and liveliness of motor power. Dr. Arnold has not been able to satisfy himself, whether there be any unequivocal evidence that mechanical irritation of the ganglia causes contractions of the intestines. In one experiment, such irritation appeared to cause contractions: on several other occasions no such effects followed. Müller, however, informs us that he distinctly observed peristaltic excitement from irritation of the cæliac ganglion; and observes that since the splanchnic nerve is the medium of communication between the sympathetic and the cæliac ganglion, while the cæliac ganglion, on its part, is connected with the spinal nerves, and through them with the spinal cord; hence irritation of the splanchnic nerve may be communicated either immediately or through the medium of the spinal cord, *to the abdominal muscles;* and hence the contractions of these muscles sympathetically and consecutively of emetic irritation of the stomach: the only share which the sympathetic has, in the production of vomiting, consisting, according to Müller, "merely in its conveying, like all other sensitive nerves the impression made upon it to the sensorium."

Some observations and experiments seem to confirm and some to discountenance the alleged fact that irritation of the vegetative nervous system may be the cause of anti-peristaltic phenomena, and of vomiting. In inflammation of the semilunar ganglion, Lobstein, Schwann, and others have noticed the occurrence of excessive pain towards the vertebral column and deep in the hypocondres, accompanied with vomiting: which phenomenon was, however, several times absent.

At page 164, et seq., Dr. Arnold considers the "*external* influences on the organism," which may give rise to vomiting. These influences principally consist of those causes which produce alterations in the condition of the stomach and intestines, or modify the action of the diaphragm and abdominal muscles. In this list are noticed tumours in the gullet, (the vomiting in this case, he would partly account for from the connexion of the fifth pair with the ganglionic nerves;) undue distention of the stomach, as stimulating the diaphragm and abdominal muscles to contract acrid aliment. Under this last head, Dr. Arnold mentions the case of a man, with whom, from idiosyncrasy, milk never agreed, and who lost his life by an imprudent use of it, when an adult.

Medicines and poisons are fruitful causes of vomiting; and Dr. Arnold remarks, in respect of the latter, that occasionally the organism, by a sort of instinctive vigilance, rejects such substances; and that too in cases, in which the article swallowed, does not *therapeutically* dispose to vomiting. This notion we regard as fanciful. (p. 171.)

Miasmatic and contagious effluvia often cause states of the organism which have vomiting for a symptom. The former species, as exemplified in ague, more particularly affects the biliary system, the spleen, and the abdominal organs generally. Contagious disease is often characterized by the same symptom and that too in cases in which the gastric organs are not peculiarly the seat of affection. In such instances, Dr. Arnold would impute the phenomenon to " irritation or alienation of the vascular or nervous systems, or of some organ in sympathetic relation to the stomach." (p. 173.)

Often in miasmatic and contagious diseases the vomiting is a sign that the organism is striving to free itself from the depressing influence of the morbific agent; and the disposition to vomit, if encouraged by art—as it always ought to be in such cases—often avails to give a milder character to the impending or commencing malady, sometimes to cut it short. But often also the vomiting in such cases is but one of the many signs of general disturbance.

Bitter, insipid, or disgusting substances, especially animal, often cause vomiting; of the final cause of which, in such cases, we are ignorant. The author informs us that in former times rasped human nails were used as an emetic, (p. 174.) The emetic effect in this case must, one would think, have been wholly due to *emotional* disgust.

Certain movements and postures of the body and the peculiar motions of a sailing-vessel may produce vomiting. As regards vomition from the last cause Dr. Arnold enters into some detail. He points out that the effect is produced sooner and in a more pronounced manner on a sea of long and gradually swelling waves than on rough, abrupt, and broken water. And this fact, he conceives, is opposed to the theory of those who would explain the phenomenon from *mere* agitation or shaking (erschütterung) of the head. Neither can it be accounted for solely or chiefly on the supposition of determination of blood to the brain—of the occurrence of which there is little or no proof. Against the notion that sea-sickness is entirely owing to a peculiar affection of the visual organs, caused by constant changes in the position and appearance of the surrounding objects and landscape, militates the fact that blind persons are as subject to the affection as those who see. Even if the inferences now referred to be allowed to have some subordinate influence in the effect, it is clear that they are not the principal causes. The actual and chief cause of sea-sickness lies in a peculiar affection of the corpora quadrigemina. According to Burdach's experiments, the corpora quadrigemina are the part of the brain from affections of which, more than from those of any other portion, the phenomenon of vomition ensues, and he admits that as the visual nerves, affected by the incessant change of objects, may propagate their temporarily morbid state to the corpora, the chain of sympathetic actions, thus formed, may be allowed to account, in part, at least, for sea-sickness in the case of those who possess vision. But as blind persons are not less liable to the affection, he explains the sickness in their case on the ground that the corpora quadrigemina being one of the chief organs of the bodily movements, the continual efforts of persons on board ship to maintain their equilibrium must produce some peculiar affection of these bodies; and since these, of all parts of the cerebral hemispheres, have the most important *psychical* influence in digestion (psychische moment) and regulate the general sensibility and the assimilative process of the digestive organs, it is easy to account, from the disturbed state of the corpora quadrigemina, for the consequent and corresponding condition of the stomach. Moreover, no part of the brain, except the fornix, is more directly concerned than are the corpora quadrigemina in the production of vertigo, which so conspicuously accompanies sea-sickness—another proof, in Dr. Arnold's apprehension, of the correctness of this theory. Dr. Budge's explanation of sea-sickness is somewhat vague and unsatisfactory. (Budge, pp. 187-9.) Müller has some very brief and general speculations on the subject of vertigo, and makes the following singular remark; "It is possible, however, that the revolving motion of the body may cause an aberration of a more subtile principle than the particles of the cerebral substance of blood; in fact, an aberration of the nervous principle itself, such as affects the sensorium, so as to produce the apparent motions of objects." (Bayly's translation, vol. i. p. 848.) There is some obscurity in this observation, and the only comment we make on it is, that the eminent author unphilosophically assumes what he cannot prove, namely, the existence of a nervous principle distinct from the cerebral substance. That there *may* be such a principle cannot be denied; but just as little can it be proved that there *is* such a principle: and in such a state of matters, therefore, the negative view is at once the more becoming and the more scientific.

*Physiological effects of vomiting.* The second part of Dr. Arnold's work is devoted to the consideration of "the changes in the organism which are connected with and which follow upon vomiting." (p. 181.)

As regards the influence of vomiting on the digestive organs, it is to be observed that when the act is what may be called idiopathic, not symptomatic of any ulterior derangement, but simply an effort of the stomach to disburden itself of, for example, indigestible aliment, the relief obtained is usually unmixed, and unequivocal. But in other cases, and sometimes even in the case referred to, there accompanies and follows the act a feeling of anguish, weakness, or an exalted sensibility of stomach. The natural vigour of the digestive function may receive a temporary prostration. This is more remarkably the case where the exciting cause has greatly deranged the digestive organ, previously to having urged it into actual vomition; or when this act itself has been violent, long-continued, or frequently recurrent. The favorable signs attending or rather following vomition are, disappearance from the tongue of any morbid coating; from the mouth of an insipid, slimy, bitter, sour or unnatural taste; cessation of the vomiting itself, preceded by the œsophageal ejection of the exciting cause, such as morbid secretions or indigestible aliment; with return of the relish for food, and ability to retain and digest the same without inconvenience.

The continuance and violence of the vomiting, which depends on incipient cerebral disease, added to a loaded tongue, ill taste, deficient appetite, but, above all, the occurrence of gastric and biliary symptoms, soon reveal to the observant physician the formidable disease with which he has to contend, and of which the vomiting is but a symptom. Ileus often seems to occur *after* energetic vomiting, and is accordingly apt to be mistaken for a consequence of it; but the order of relation is just the contrary. The same remark applies to hernia, hypertrophy of the stomach, and several other lesions. It may be here noticed, that in vomition of this violent character, the stomach may be lacerated; of which accident cases are recorded by Boerhaave, Guersent, Bouillaud, Andral, &c. This grave catastrophe is usually preceded by ramolissement. Sometimes the lower part of the œsophagus is the seat of rupture. In the one case, effusion of the stomachic contents takes place into the pleural cavity; by pressure of which on the lungs or heart, life may be instantaneously extinguished. In the other case, the stomachic contents pass into the peritoneum. In either case, inflammation destroys the patient, in the event of life lasting so long as to allow of this process being set up.

Severe and protracted vomiting sometimes gives rise to anti-peristaltic action of the intestines, terminating in the ejection upwards of fecal matters. The more usual effect of vomiting on the intestinal canal is simply an increase of the alvine secretions and stools. The sudden augmentation of saliva, during nausea and vomiting, Dr. Arnold says, is "perhaps owing to the determination of blood to the head." The biliary and pancreatic fluids flow in profusion partly owing to the pressure of the abdominal and diaphragmatic muscles. All the secretions likewise lose something of their usual spissitude in the course of vomiting.

In noticing the effect of vomiting on the womb, Dr. Arnold states that certain emetics, among which ipecacuan is enumerated, are apt to produce hemorrhage from that organ.

During the retention of the breath, which immediately precedes the act of vomiting, both the lungs and heart are apt to suffer: congestion or apoplexy of the former, rupture or dilatation of the latter may occur: though this chiefly or only, where there is predisposition to such accidents from the presence of previous disease. In the violent expiratory act, which instantly follows the retention of breath just alluded to, the contents of vomicæ and foreign bodies lodged in the lungs or bronchiæ, may be ejected with great violence.

*Therapeutical effects of vomiting.* The therapeutic and dietetic employment of vomiting is ancient. The Egyptians were accustomed to practise it as a means of inward purification. The luxurious Romans freqnently took emetics before meals; and also immediately after them, in order that, with safety and renewed zest, they might recommence the feast. If we remember aright, Cæsar, being invited to spend a day with Cicero, at one of his suburban villas, took an emetic before dinner, as a civil mode of testifying his resolution to do ample justice to the entertainment provided for him by the immortal orator. It would appear from an observation of Dr. Arnold, that this bad habit is beginning to prevail among the academic youth of his own country, (p. 212,) to whom, he informs us with regret, the words of Cicero may be applied: "Ab horâ tertia, bibebatur, ludebatur, *vomebatur!*"

Our own Fothergill was very partial to the use of vomiting as a dietetic means: and thought that judicious recourse to it, at proper intervals, might contribute to prolong life. But the most remarkable eulogist of vomiting is Mentzel: who singularly enumerates atrophy as among the diseases which systematic vomition is fitted to avert; Bremser, on the other hand, observes with greater justice, that a frequent use of emetics, by enervating the stomach, cannot fail to induce general debility; and if so, it is not easy to see how longevity can be secured or promoted by the employment of them.

We suspect that the use of emetics, in the case of substances (more especially of a pointed form, as a fish-bone, &c.,) being impacted in the pharynx or œsophagus, is problematical; even supposing it to be possible to administer emetics in such circumstances. In the cases of this description which have come under our notice, vomitive efforts appeared to be rather prejudicial than the contrary, by engaging the foreign substance more deeply in the soft parts. However, in some particular cases, the plan may be worthy a trial.

When poison has been swallowed, especially of an acrid or narcotic nature, prompt evacuation of the stomach is obviously necessary; but care must be taken that the emetic does not merely add to the mischief already inflicted. Tickling the fauces is a safe plan; introducing the stomach-pump is equally safe, and more efficient, when the poisonous *ingestum* has been fluid; but when it is solid, emetics are needed. Orfila gives, in the first place, two or three grains of tartar emetic, with from twenty to thirty grains of ipecacuan; and after waiting for some time, (the first dose, we presume, having been unsuccessful,) he gives a second, consisting of the same quantity of tartar emetic combined with an ounce or an ounce and a half of Glauber salts, in order to carry down the stomachic contents into the intestines and compel their evacuation in this manner.

Dr. Arnold justly observes that in cases of redundant mucous secretion of the stomach and bowels, the benefit to be derived from emetics is temporary, not radical or complete; such discharges depending on a morbid state of the mucous membrane. Vomiting, whether induced by nature or art, serves merely to disembarrass the stomach, for a time, of the slimy profluvium; but other treatment is required in order to a perfect removal of the disease, which more usually depends on irritation than relaxation. He mentions the hydrochlorate and tartrate of ammonia as the most useful emetics in cases of slimy mucus, from the property which these salts possess of dissolving the secretion; but they are not to be resorted to in inflammatory states of the stomach.

The influence which vomiting has on various other affections of the stomach, bowels, and adjacent viscera, as the liver, spleen, lungs, kidney, nervous system, and organs of sense, is set forth with amplitude and sufficient accuracy and intelligence. There are, however, in this part a considerable degree of diffuseness and an unnecessary reproduction of information already familiar to all moderately informed practical physicians. For these reasons, and in order to keep within due limits, we must pass over, without notice, this part of the volume, and proceed to the fifth section, in which vomiting is considered in its therapeutical and pathological aspects, and the best means are pointed out, now of exciting and now of alleviating it.

Mere motion will, as has been already remarked, give rise to vomiting, and may therefore be occasionally selected as a means for producing the same. Thus, certain movements in a bed, hammock, or swing create nausea and vomition. Rotatory motion is an effectual means, and in this case the sickness follows much sooner should the patient be standing or sitting than in the horizontal position. Now vomition may in this manner be usefully induced in cases in which a rapid effect is desirable or indispensable—as, for example, when poison has been taken; but in which the sensibility of the stomach is injured. It is useful also, according to Esquirol, in mental affections, which are preceded by signs of great vitiation of the gastric secretions.

Sea-sickness is peculiarly adapted for the milder mental affections, as hypochondria; and among corporeal derangements, for swellings of the lymphatic glands.

Tickling the fauces, &c. is suited, as a vomitive means, for cases in which the stomach is very much distended, and a disposition to vomit either already exists or may be easily excited. It is to be had recourse to also when an emetic cannot be swallowed or else cannot be obtained, yet in which the necessity for it is urgent, as in cases of asphyxia or poisoning. It is a means, however, not to be depended upon, and which seldom fully effects the desired end.

Insipid drinks, such as lukewarm water, sweet or oily fluids, &c. are to be used rather as a subordinate means of facilitating, increasing, and prolonging vomition induced by other means, than to be employed as independent ones.

*Medicinal emetics.* Emetics are derived from the three kingdoms of nature, but principally from the mineral and vegetable. Antimony is the principal emetic from the former source, and is in general effectual. When not so, and when incautiously administered in an overdose, it may prove fatal. It is indicated as an emetic—1st. In the case of strong but not too excitable subjects, and in persons not too debilitated by age or disease. 2d. In cases in which prompt and powerful vomition is desired, and in which the subsequent evacuations from the bowels which may possibly follow are not dreaded as too debilitating. 3d. In cases in which it is an object to lessen the spissitude of the secretions, whether of the alimentary or respiratory passages, and of the tegumentary surface, and to maintain these in an active state; also to forward the appearance of febrile eruptions. 4th. To remove obstructions of or torpor in the lymphatic and glandular systems, and to stimulate the function of absorption. 5th. In some cases of inflammation and hyperemia tartar emetic is useful, not only by disembarrassing the stomach and emptying the vascular system, but, when given in small doses, by its peculiar effect on the inflamed organs, provided they be such as owe their sensibility to the vagus.

Sulphate of zinc is praised (p. 370) for its efficacy in detaching false membranes in the air-passages; an effect which tartar-emetic and ipecacuan often fail of accomplishing. It is adapted to cases of apoplexy, asphyxia, and mental disease, and will often excite vomiting when poison has been taken, and when the stomach, from reduced sensibility, resists the action of other emetics.

The preparations of copper constitute extremely prompt and efficacious means of inducing vomiting. The union of sulphate of copper and tartar-emetic is affirmed (p. 373) to be extremely efficacious in thoroughly emulging the biliary receptacles, without debilitating the digestive organs.

Ipecacuan is the chief emetic from the vegetable kingdom. The indications for its employment are stated to be, a tendency to diarrhœa, with nausea and other circumstances, as a foul tongue, &c. suggesting the propriety of an emetic. 2d. Great debility, by which stronger emetics—as antimony—are contra-indicated: spasms. 3d. Hemorrhage from the rectum, uterus, lungs, or other organs, in which the revulsion of an emetic is likely to be useful.

Other emetics are named, as iris-root, asarum, white hellebore, &c., used by the ancients, but now never employed.

Few or no substances from the animal kingdom are ever had recourse to as emetics. The eggs of the barbel are said to act as such. According to Ettmüller, the eggs of ants have a similar effect. Dr. Arnold tells us (p. 388) that "rasped human nails digested in wine, and taken after being filtrated, excite energetic stools and vomiting!"

In administering emetics the state of the vascular system must be considered; and in the case of plethoric persons and those predisposed to apoplectic seizures, venesection must be premised. When the sensibility of the stomach is greatly reduced, or vital power is low, brandy taken along with or subsequently to the emetic will facilitate the operation of it. The same effect will be produced in like subjects by a blister to the epigastric region, or hot epithems there. With some excitable persons the administration of opium on the night previous to the taking of the emetic is almost the only means of ensuring the operation of the latter. The exhibition of emetics in clyster is an uncertain and awkward method. Schwerer asserts that five grains of tartar-emetic, dissolved in warm water and held in the hand, will produce emesis. According to Cox, a moist tobacco-leaf laid on the epigastrium will do the same. There are, no doubt, many occasions when it might be desirable to bring about vomition in this indirect manner, by which the general vigour of the patient, and the strength of the digestive organs in particular, would be spared. We all know that vomiting may be produced by the introduction of a solution of tartar-emetic into the veins. Perhaps the deglutition of tobacco-smoke might be made to answer the end.

But we must pause here: and the length to which our remarks on Dr. Arnold's volume have extended preclude us from entering on an examination of the extremely interesting, but perhaps less accurate, connected, methodical, as well as less complete treatise of Dr. Budge. Both works are in the highest degree creditable to the medical literature and science of Germany; and furnish the best possible evidence of the zeal and talent which characterize our professional collaborators in that part of the Continent.

---

